# Novel therapeutic target for diabetic kidney disease through downregulation of miRNA-192-5p and miRNA-21-5p by celastrol: implication of autophagy, oxidative stress, and fibrosis

**DOI:** 10.1007/s00210-024-03669-5

**Published:** 2024-12-19

**Authors:** Samar. M. Al-Tantawy, Salma.M. Eraky, Laila.A. Eissa

**Affiliations:** Department of Biochemistry, Faculty of Pharmacy, Mansoura University, Mansoura, 35516 Egypt

**Keywords:** Celastrol, Diabetic kidney disease, MiRNA-192-5p, MiRNA-21-5p, TGF-β1, Transforming growth factor β1

## Abstract

One of the most common microvascular effects of diabetes mellitus (DM) that may result in end-stage renal failure is diabetic kidney disease (DKD). Current treatments carry a substantial residual risk of disease progression regardless of treatment. By modulating various molecular targets, pentacyclic triterpenoid celastrol has been found to possess curative properties in the treatment of diabetes and other inflammatory diseases. Therefore, the present study investigated whether celastrol has anti-inflammatory, antioxidant, and antifibrotic effects as a natural compound against experimental DKD. Streptozotocin (55 mg/kg) was utilized for inducing DKD in a rat model. Antioxidant enzymes and renal function tests were assessed in serum samples. In kidney homogenate, relative miRNA-192-5p and miRNA-21-5p gene expressions were measured. Furthermore, using real-time PCR to evaluate the gene expressions of nucleus erythroid 2–related factor-2 (Nrf-2), matrix metalloproteinase-2 (MMP-2), proapoptotic caspase-3, antiapoptotic Bcl-2, LC-3, and Beclin-1. Moreover, the transforming growth factor β1 (TGF-β1), LC-3, Bcl-2, caspase-3 and NADPH oxidase 4 (NOX4) renal expressions were assessed semi-quantitatively using immunohistochemistry. Seven weeks of celastrol (1.5 mg/kg/day) treatment significantly ameliorated DKD. Celastrol improves kidney functions. Moreover, celastrol treatment demonstrated potent antioxidant effect. The mechanism of apoptosis resulting from the administration of celastrol included the modulation of Bcl-2 and caspase-3 expression in the kidney. Celasterol administration leads to an increase in LC-3 and Beclin-1 renal expression that resulting in autophagy. Celastrol treatment improved renal fibrosis by decreasing TGF-β1 and MMP-2 renal expression. These antifibrotic effects could be due to their ability to inhibit miRNA-192-5p and miRNA-21-5p expression in renal tissues. Celastrol exerts a renoprotective effect by targeting miRNA-21 and miRNA-192, as well as their downstream pathways, such as autophagy, apoptosis, and fibrosis.

**Supplementary Information:**

The online version contains supplementary material available at 10.1007/s00210-024-03669-5.

## Introduction

Diabetes mellitus (DM) is a systemic illness characterized by the body’s incapacity to produce enough insulin or respond to it appropriately. The blood flow and metabolic homeostasis are upset by the ensuing rise in blood glucose levels. Furthermore, the persistent imbalance in the microenvironment fosters the development of widespread cellular abnormalities. Due to their high sensitivity to hemodynamic and metabolic changes, kidneys are vulnerable targets in diabetic patients (Fu et al. 2019). DKD is a severe microvascular DM complication (Lin et al. 2018). It is high blood pressure as well as albuminuria, finally leading to end-stage renal disease (ESRD) (Samra et al. 2016). Controlling hyperglycemia and blood pressure comprises the current therapy approach for DKD. Nevertheless, patients still experience extensive kidney damage. Consequently, it is necessary to search for novel treatment strategies (Wang et al. 2014).

Oxidative stress is a significant factor that links hyperglycemia to vascular complications through metabolic changes in target tissue molecules and renal hemodynamics (Sifuentes-Franco et al. 2018). NADPH oxidase 4 (NOX4) plays a central role in ROS production, particularly in DKD, where hyperglycemia-induced NOX activation contributes to oxidative damage in renal tissues, thereby exacerbating fibrosis and cellular apoptosis. Consequently, targeting NOX activity offers a promising approach to mitigate oxidative stress-induced complications in DKD (Lee et al. 2020). Two types of cell death that contribute to the formation of DKD include autophagy along with apoptosis. Autophagy is important to preserve cell homeostasis in stress conditions like hypoxia and oxidative damage. Autophagy impairment in proximal and podocyte tubular cells has been linked to DKD pathogenesis (Erekat 2022).

Apoptosis is the event of natural cell death mandatory for multicellular organisms’ homeostasis and normal development. Apoptosis occurs in epithelial cells and podocytes of proximal convoluted tubules along with DKD progression. Bcl-2 is one of the essential antiapoptotic proteins, while Bax is a proapoptotic regulator (Borkan 2016). Caspase-3 acts as a proapoptotic protein and contributes to the transmission of apoptotic signals to the endoplasmic reticulum (Sha et al. 2017).

Beclin-1, which is known as a substantially conserved eukaryotic protein, functions as a critical regulator that mediates vesicle trafficking and thus stimulates autophagy. Beclin-1 is a phosphatidyl inositol-3-kinase (PI3K) crucial component which stimulates autophagy (Naguib and Rashed 2018). The light chain 3 (LC3) protein, which is a human equivalent of the yeast autophagy-related gene 8 (Atg8), has been discovered as a particular marker for autophagy. It relates to microtubules. During the initiation of autophagy, the protein LC3 binds with phosphatidylethanolamine and is positioned on autophagic membranes (Kuma et al. 2007). Single-strand, short-range RNAs targeting more than 50% of protein-coding transcripts and non-coding when combined is known as microRNAs (miRNAs). Recent research has demonstrated that microRNA contributes to several human disease pathogenesis, like diabetes and its complications (Greco et al. 2020). Under hyperglycemic conditions, altered expression of miRNA-21-5p has been found to affect inflammatory and fibrotic marker production (Kaur et al. 2022). It was reported that microRNA-192-5p could enhance DKD progression by modulating the signaling pathways of TGF-β1 (Saadi et al. 2019).

From the small segment of triterpene quinine methides, celastrol is a pentacyclic triterpenoid. Furthermore, it is a monomeric chemical compound that was isolated from *Tripterygium wilfordii*, a Chinese herb (Cascão et al. 2017). Based on its anti-inflammatory and immunomodulatory properties, celastrol has demonstrated potent efficacy in treating hyperglycemia and chronic kidney disease, as evidenced by several studies (Wang and Zeng 2019; Venkatesha and Moudgil 2016; Nie et al. 2020). Another study (Tang et al. 2019) also reported that celastrol had an antifibrotic effect throughout renal fibrosis progression.

This research aims to examine the potential celastrol renoprotective impact, along with the potential underlying molecular pathways (with a focus on miR-21-5p and miR-192-5p), and autophagy, including oxidative stress and its their downstream pathways. Fibrosis and apoptosis are also to be considered.

## Methods and materials

### Drugs and chemicals

Sigma-Aldrich (St. Louis, USA) provided both STZ (purity > 98.0%, No. 188883–66-4) and celastrol (purity > 99.9%, No. 34157–83-0).

### Animals

The Faculty of Pharmacy’s ethical committee at Mansoura University approved this study’s animal protocol with approval number 2023–134. A total of 30 male Sprague Dawley rats, with an average weight of 200 ± 20 g, were provided with unrestricted access to tap water and food. Standard conditions established were 12-light/12-dark cycles and a 22 ± 2 °C temperature.

### Induction of diabetes

Following an acclimatization for 1 week and fasting for 12 h, the rats were intraperitoneally injected with STZ dissolved at a dose of 55 mg/kg in citrate buffer (0.1 M, pH = 4.5) in order to induce diabetes (Tang et al. 2019). After inducing diabetes in rats for 48 h, the rats’ blood glucose level was calculated starting from the tail vein with a glucometer.

### Experimental design

Animals were categorized into the following three different groups: rats in group 1 (control; *n* = 8) received a single dose of citrate buffer (0.1 M, pH 4.5) intraperitoneally. Rats in group 2 (DKD group; *n* = 12): As previously demonstrated, rats were injected with STZ to induce diabetes. Rats in group 3 (DKD + celastrol group: *n* = 10) were administered celastrol (1.5 mg/kg/day) diluted in saline containing 5% dimethyl sulfoxide (DMSO) intraperitoneally for 7 weeks (Abdelaty et al. 2020).

### Sample collection as well as processing

After 7 weeks of the trial, the rats were subjected to weighing, and using metabolic cages, 24-h urine samples were taken from them. The urine specimens had been spun after that and then utilized shortly thereafter for biochemical analysis. 40 mg/kg IP of sodium pentobarbital was to put rats asleep, used. Rats’ blood was stored at − 20 °C for further biochemical examinations after drawn using retro-orbital punctures, allowed to clot, then aliquoted and processed. The animals were scarified, and their kidneys were extracted out and immediately cleaned using ice-cold saline. Next, the kidneys were weighed. The weight of the kidney is divided by the body’s total weight to get the kidney weight index. After dividing the right kidney in half, the half that was not used for further histological and immunohistopathological analysis was placed in 10% phosphate-buffered formalin. The second component was promptly frozen and stored (in liquid nitrogen) for the miRNA and rapid PCR assessments.

### Biochemical analysis of serum

The level of glycated hemoglobin (HbA1c) in the serum of rats was determined using a rat HbA1c ELISA Kit (Cusabio, China) following the instructions provided by the manufacturer. Furthermore, the serum was subjected to measurements of catalase (CAT), total antioxidant capacity (TAC), and malondialdehyde (MDA) using commercially available colorimetric assays in accordance with the manufacturer’s instructions. Colorimetric kits from Biodiagnostic, Giza, Egypt, were used to measure the levels of creatinine and serum urea.

### Biochemical analysis of urine

First, ﻿24-h total protein in urine and 24-h total levels of creatinine were measured utilizing colorimetric kits obtained from Biodiagnostic, Egypt, before being utilized for the determination of creatinine clearance based on the equation as follows:

“Urinary creatinine level (mg/dL) divided by urine flow (mL/min) equals creatinine clearance (mL/min). Serum creatinine level (mg/dL),” Urine flow was determined by dividing the 24-h urine volume by the total number of minutes in a day (1440 min) (Bazzano et al. 2015). Additionally, microalbuminuria measurement was performed utilizing Rat Microalbuminuria ELISA Kit (Elabscience, USA) as per the manufacturer.

### MicroRNA assay for miRNA-21-5p and miRNA-192-5p

Extraction of total RNA from renal tissues, such as miRNA, from renal tissue was done utilizing miRNeasy Mini Kit (Qiagen, MD, North America) following the manufacturer. Following the manufacturer’s instructions, the miRNA was reverse transcribed to cDNA using the starting Kit from Qiagen, USA. Instant PCR amplification was then performed using enhanced primers with LNA locked into nucleic acids. The U6 gene was used as a reference for internal control.

### mRNA gene expression assays

A publicly available equipment (Thermo Scientific, USA) was used in accordance with the manufacturer’s instructions to turn 5 g of RNA samples into DNA that is complementary (cDNA). Subsequently, RT-PCR was performed using cDNA and Thermo Scientific Maxima SYBR Green QPCR Master Mix (2x) from Thermo Fisher Scientific Inc. (USA). Table 1 indicates that the primer sequence was acquired from Vivantis Company in Malaysia. The RCN of the target genes (Nrf-2, MMP-2, caspase-3, Bcl-2, Beclin-1, and LC-3) was calculated by comparing their Ct values to HPRT-1. The 2^−ΔΔCT^ method was also used to determine the proportion of each component of the amplification of gene product.

**Table 1:** Primers’ sequences of all studied genes

Gene	Direction	Sequence	Reference sequence	Product size
Bcl-2	Forward	5′-TGTGTGGAGAGCGTCAACAG-3′	NM_016993.2	175
	Reverse	5′-ACAGCCAGGAGAAATCAAACAG-3′		
Caspase-3	Forward	5′-GTGGAACTGACGATGATATGGC-3′	NM_012922.2	135
	Reverse	5′-CGCAAAGTGACTGGATGAACC-3′		
Nrf-2	Forward	5′-TTTGTAGATGACCATGAGTCGC-3′	NM_031789.2	142
	Reverse	5′-TGTCCTGCTGTATGCTGCTT-3′		
MMP-2	Forward	5′-CCAACTACAACTTCTTTCCCC-3′	NM_031054.2	114
	Reverse	5′-GAGCAAAGGCATCATCCAC-3′		
LC-3	Forward	5′-GCGATACAAGGGTGAGAAGC-3′	NM_022867.2	187
	Reverse	5′-CACTTCAGAGATGGGTGTGG-3′		
Beclin-1	Forward	5′-CTGAGGAATGGAGGGGTCTA-3′	NM_001034117.1	159
	Reverse	5′-GCCTGGGCTGTGGTAAGTAA-3′		
HPRT-1	Forward	5′-TTCCTCCTCAGACCGCTTTT-3′	NM_012583.2	79
	Reverse	5′-ATCACTAATCACGACGCTGGG-3′		

*Bcl-2*, B-cell lymphoma 2; *HPRT-1*, hypoxanthine–guanine phosphoribosyl transferase; *LC-3*, microtubule-associated protein 1A/1B-light chain 3; *MMP-2*, matrix metalloproteinase-2; *Nrf-2*, nuclear factor erythroid 2-related factor 2

### Histological assessment

The kidney tissues were rinsed and dehydrated using ethanol after being treated with formalin. Then, they were clarified in xylene and finally placed in paraffin wax for embedding. After the tissues were cut into 5-μm-thick slices, sections were stained with periodic acid-Schiff (PAS). Glomeruli were digitally photographed, and the images were imported to the ImageJ software (National Institutes of Health, Bethesda, MD, USA; https://imagej.nih.gov/ij/) and analyzed morphometrically (Yasuzawa et al. 2021).

### Immunohistochemical analysis of TGF-β1, Bcl-2, NOX4, caspase-3, and LC-3, renal expression

Rabbit LC-3, TFG-β1, Bcl-2, NOX-4, and caspase-3 polyclonal primary antibodies (Chongqing Biospes, China) were used based on standardized immunohistochemistry protocols, followed by sections’ deparaffinization and rehydration. For endogenous peroxidase inhabitation, a hydrogen peroxide solution was used. Slide incubation at 37 °C was performed for 1 h using the primary antibodies. Following the washing step, a secondary antibody that is specific to the species was administered and allowed to incubate at a temperature of 37 °C for duration of 1 h. The staining agent was used as a counterstain, and an evaluation of antibody binding was conducted using a diaminobenzidine kit. Ultimately, the slides underwent examination using a light microscope to detect the presence of caspase-3, Bcl-2, TFG-β1, NOX-4, and LC-3 in the kidney tissues.

### Statistical analysis

The analysis of the data was conducted via SPSS software version 24, and the results were presented as Mean SE. The normality of the variables was assessed using the Shapiro–Wilk test. To examine group differences, the ANOVA with LSD post hoc testing was utilized. Employing Dunn’s post hoc and Kruskal–Wallis tests for non-parametric data, the histopathological values were investigated. The graphs were generated using GraphPad Prism software version V 6.01 (GraphPad Software Inc., San Diego, CA, USA). A probability statistic of less than 0.05 was used in determining the statistical significance level.

## Results

### Impact of celastrol on the weight index of the kidney/body

Compared to controls, the DKD group displayed a marked weight index elevation in the kidney/body, reaching 0.74 folds. Treatment with celastrol significantly decreases kidney/body weight index by 0.3 folds (Table 2).

**Table 2:** Effect of celastrol on the kidney/body weight index and kidney function parameters

Groups	Control	DKD	DKD + celastrol
Kidney/body weight index (× 10^3^)	4.3 ± 0.16	7.5 ± 0.8^a^	5.3 ± 0.32^ab^
Creatinine (mg/dL)	0.53 ± 0.04	0.82 ± 0.03^a^	0.68 ± 0.01^ab^
Urea (mg/dL)	20.71 ± 0.52	24.67 ± 1.05^a^	19.83 ± 0.54^b^
Microalbuminuria (mg/day)	17.14 ± 0.60	33.17 ± 0.87^a^	19.5 ± 1.09^b^
24-h total protein in urine (mg/24 h)	27.86 ± 0.80	191 ± 22.36^a^	48 ± 1.71^b^
Creatinine clearance (mL/min)	188.14 ± 2.74	82.9 ± 5.3^a^	161.83 ± 4.74^ab^

Data are presented as Mean ± SEM. Number of animals in each group = 8

^a^*P* < 0.05 versus the control group

^b^*P* < 0.05 versus the DKD group

### Impact of celastrol on blood glucose levels and HbA1c

**Fig. 1:**
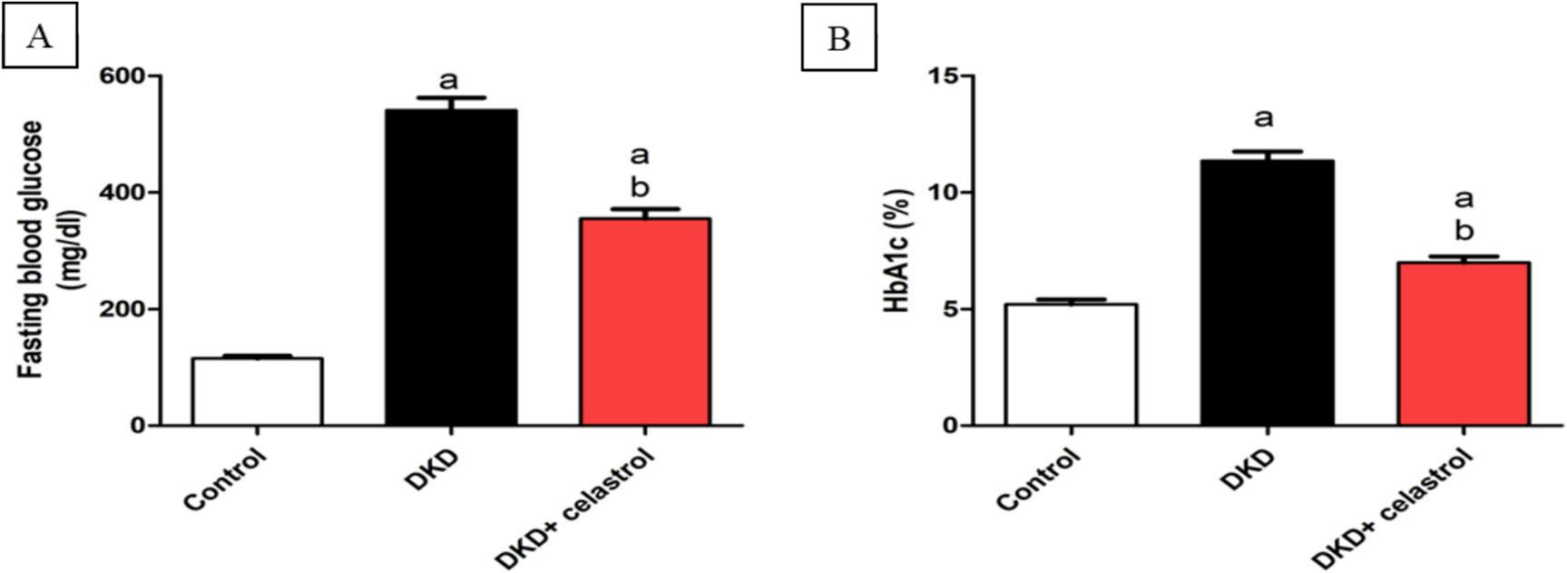
Effect of celastrol (1.5 mg/kg/day, orally) on blood glucose level (**A**) and glycated hemoglobin (HbA1c) (**B**) in a rat model of diabetic kidney disease (DKD) induced by a single intraperitoneal injection of 55 mg/kg streptozotocin. Values are presented as Mean ± SEM. Number of animals in each group = 8. a = significant at *P* < 0.05 versus the control group. b = significant at *P* < 0.05 versus the DKD group

DKD group displayed substantially elevated blood glucose levels and HbA1c reaching nearly 3.7 and 1.2 folds, respectively, compared to controls. Treatment with celastrol resulted in a significant drop in blood sugar levels and HbA1c by about 0.34 and 0.38 folds, respectively, compared to the DKD group (Fig. 1A and B).

### Impact of celastrol on renal function parameters

Compared with controls, serum creatinine, urea, microalbuminuria, and 24-h total protein levels were significantly elevated in the DKD group by about 0.55, 0.19, 0.94, and 5.85 folds, respectively. Treatment with celastrol significantly decreased creatinine, urea, microalbuminuria, and 24-h total protein levels in urine by approximately 0.2, 0.2, 0.41, and 0.74 folds, respectively, in comparison to the DKD group. Additionally, the DKD group displayed a marked decline in creatinine clearance by about 0.44 folds compared to controls. Celastrol treatment exhibited substantially increased creatinine clearance, reaching about 0.95 folds in comparison to the DKD group (Table 2).

### Impact of celastrol on oxidative stress

**Fig. 2:**
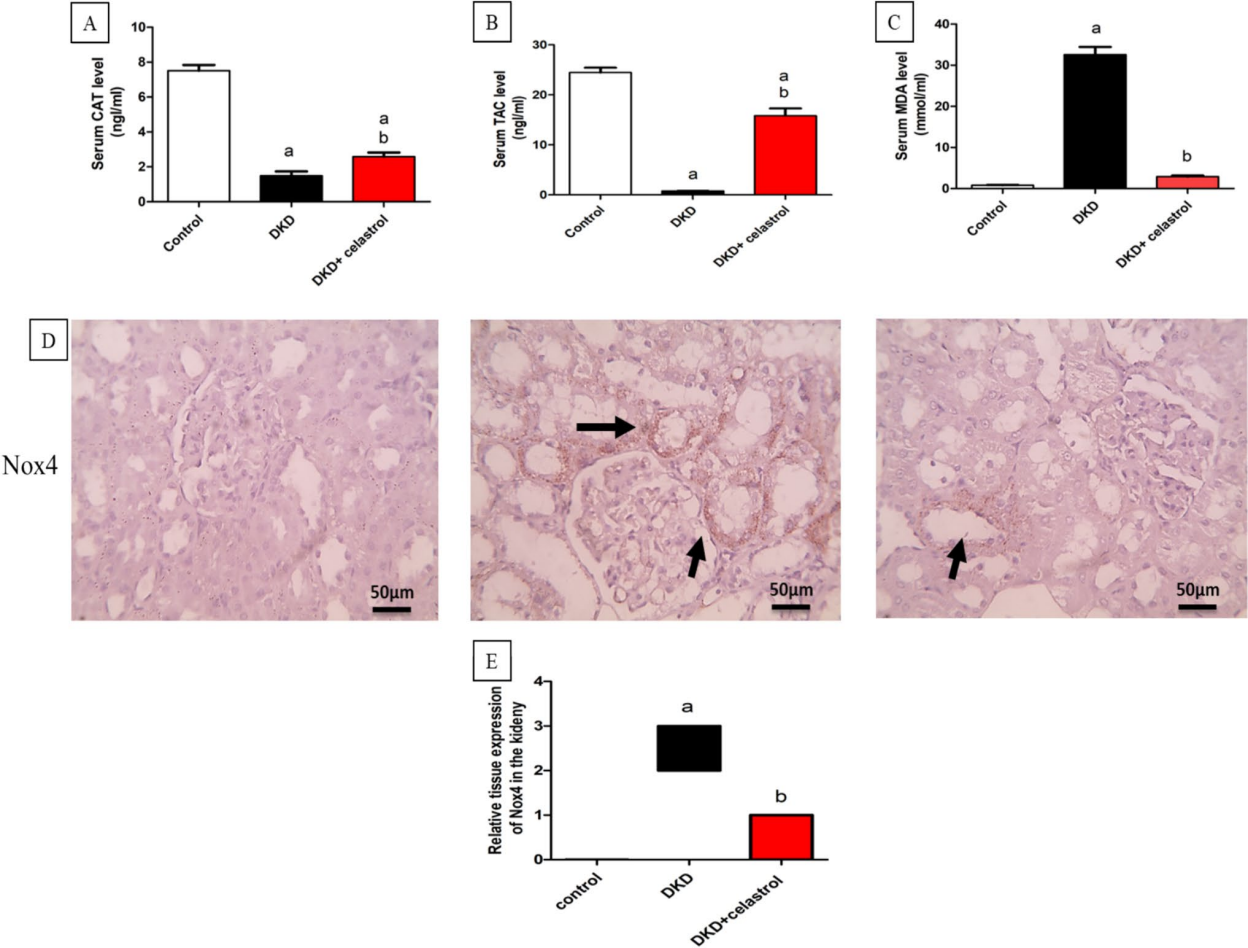
Effect of celastrol (1.5 mg/kg/day, orally) on serum catalase (CAT) (**A**), total antioxidant capacity (TAC) (**B**), and malondialdehyde (MDA) (**C**) levels in a rat model of diabetic kidney disease (DKD) induced by a single intraperitoneal injection of 55 mg/kg streptozotocin. Values are presented as Mean ± SEM. Number of animals in each group = 8. a = significant at *P* < 0.05 versus the control group, b = significant at *P* < 0.05 versus the DKD group. (**D**) Microscopic pictures of immunostained renal sections against Nox-4 showing negative expression in control group, marked positive brown tubular reaction (arrow) in DKD group, mild positive brown tubular reaction (arrow) in DKD + celastrol group. Magnifications × : 400 bar 50. (**E**) Quantitative analysis of Nox-4 relative tissue expression. Data are presented as the median of IHC score against Nox-4 analyzed by the Kruskal–Wallis test followed by Dunn’s test.

DKD group displayed substantially elevated MDA serum levels by about 37.5 folds with substantially declined CAT and TAC serum levels by about 0.8 and 0.96 folds, respectively, compared with controls. Celastrol treatment resulted in a substantial decline in MDA serum level by about 0.91 folds with a considerable elevation in CAT and TAC serum levels by about 1.38 and 19 folds, respectively, compared to the DKD group (Fig. 2A: C). The immunohistochemical study of Nox-4 validated these findings, as rats treated with celastrol exhibited reduced NOX-4 tissue expression compared to the DKD group (Fig. 2D and E).

### Impact of celastrol on the histopathological examination

**Fig. 3:**
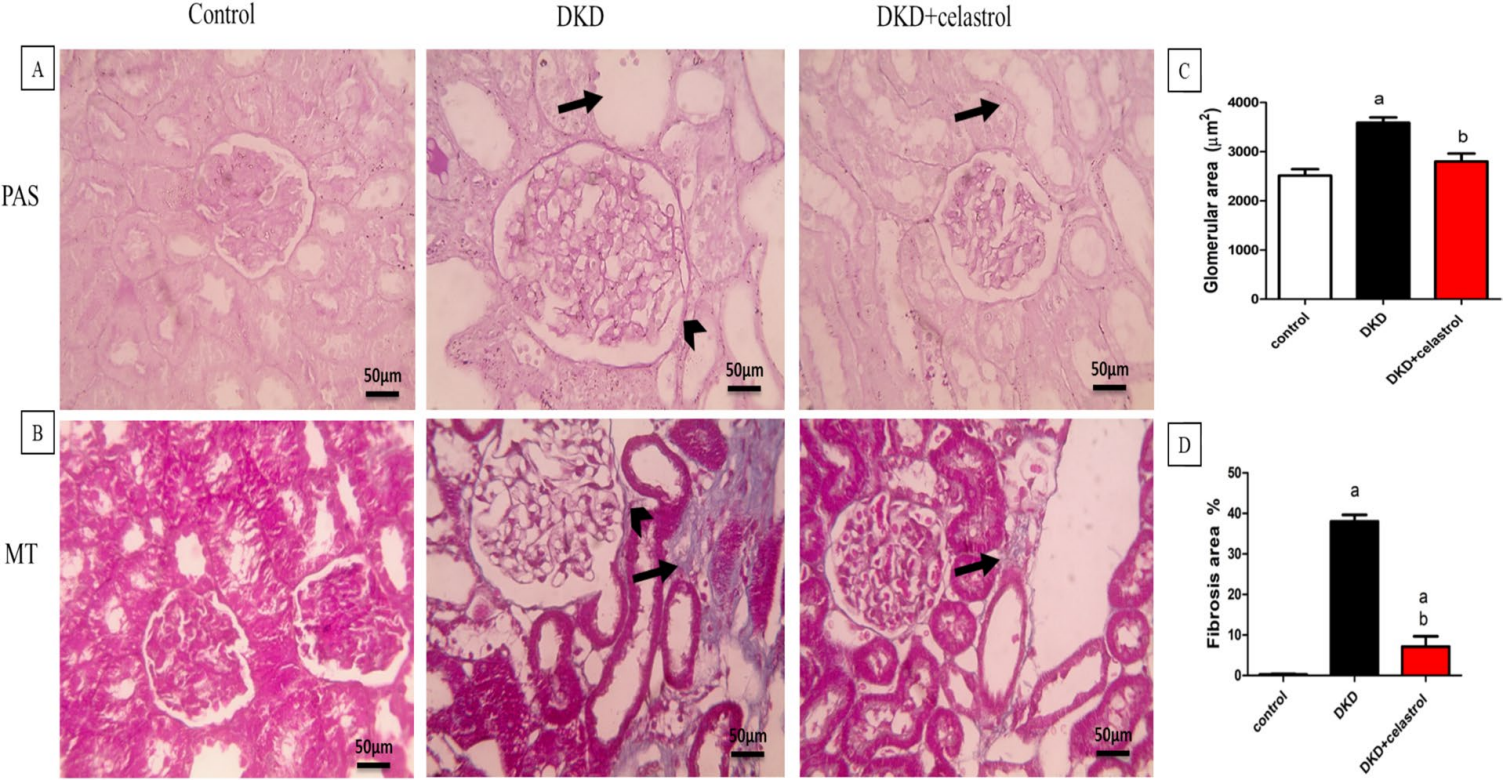
Microscopic pictures of PAS-stained renal sections showing normal glomeruli and tubules with minimal interstitial tissue in the control group. Markedly enlarged glomeruli, thickened glomerular basement membranes with expansion of Bowman’s gap (arrowhead) and severe tubular dilation (arrow) are seen in DKD group. Renal sections from DKD + celastrol group showing decrease glomerular area and mild tubular dilation (arrow) are seen. Magnifications × : 400 bar 50 (**A**). Microscopic pictures of Masson trichrome-stained renal sections showing no fibrosis in control group, excess bluish fibrosis in interstitial tissue (arrow) with thickened Bowman’s capsule (arrowhead) in DKD group, mild bluish fibrosis in interstitial tissue (arrow) with normal Bowman’s capsule in DKD + celastrol group. Magnifications × : 400 bar 50 (**B**). Bars are Mean ± SEM of glomerular area fibrosis (**C**) and fibrosis area % (**D**) and that statistically analyzed by one-way ANOVA followed by Tukey’s test. Number of animals in each group = 8. a = significant at *P* < 0.05 versus the control group, b = significant at *P* < 0.05 versus the DKD group

Renal sections stained with PAS showed normal glomeruli and tubules with minimal interstitial tissue in the control group. On contrast, DKD showed markedly enlarged glomeruli, thickened glomerular basement membranes with expansion of Bowman’s gap and severe tubular dilation. On the other hand, renal sections from treated rats with celastrol showed decreased glomerular area, decreased expansion of Bowman’s gap, and moderate tubular dilation. In addition, microscopic examination of Masson trichrome-stained renal sections revealed distinct differences between the groups. In the control group, there was no evidence of fibrosis, with the renal tissue appearing normal. In contrast, the DKD group displayed significant fibrosis in the interstitial tissue, highlighted by excess bluish staining, and a thickened Bowman’s capsule. However, in the DKD + celastrol group, there was milder fibrosis in the interstitial tissue, and the Bowman’s capsule appeared nearly normal (Fig. 3A and C).

### Effect of celastrol on miRNA-21-5P and miRNA-192-5P

**Fig. 4:**
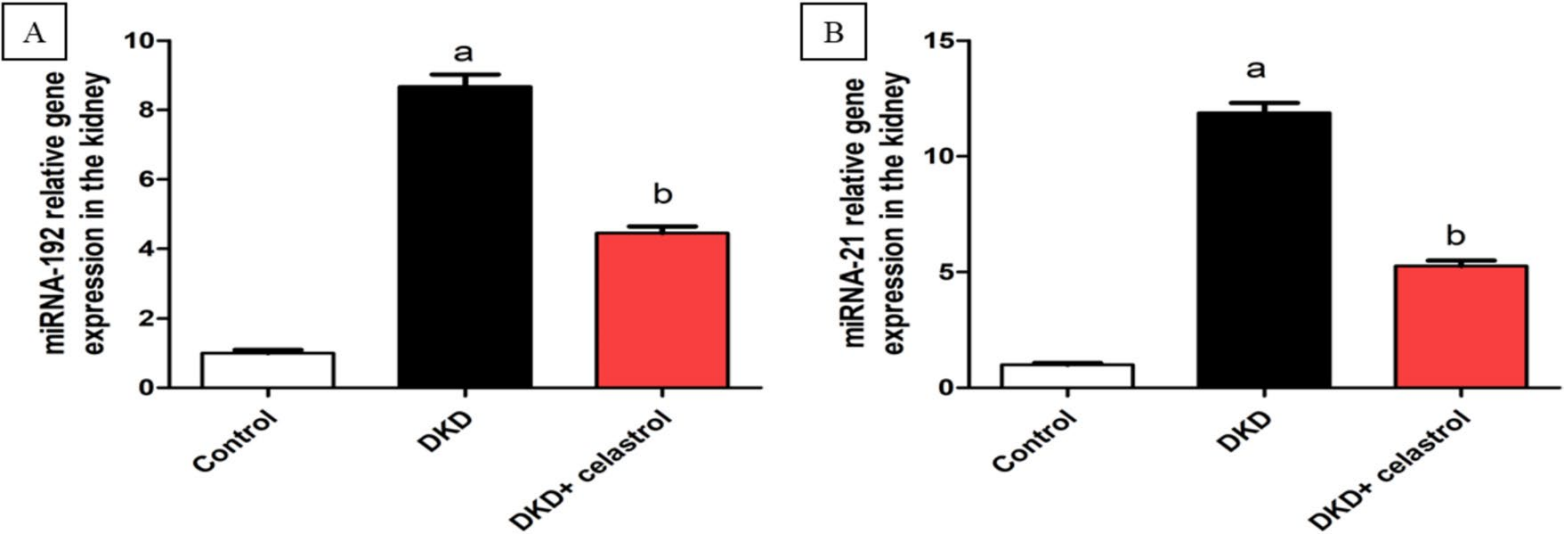
Effects of celastrol (1.5 mg/kg/day, orally) on relative gene expression of microRNA-192-5p (miRNA-192) (**A**) and microRNA-21-5p (miRNA-21) (**B**) in a rat model of diabetic kidney disease (DKD) induced by a single intraperitoneal injection of 55 mg/kg streptozotocin. Values are presented as Mean ± SEM. Number of animals in each group = 8. a = significant at *P* < 0.05 versus the control group. b = significant at *P* < 0.05 versus the DKD group

Compared with controls, miRNA-192-5P and miRNA-21-5P renal expression was substantially increased in the DKD group by about 7.7 and 10.88 folds, respectively. Celastrol treatment significantly decreased miRNA-21-5P and miRNA-192-5P by approximately 0.5 folds and 0.48, respectively, compared with the DKD group (Fig. 4A and B).

### Celastrol impact on renal expression of apoptotic markers

**Fig. 5:**
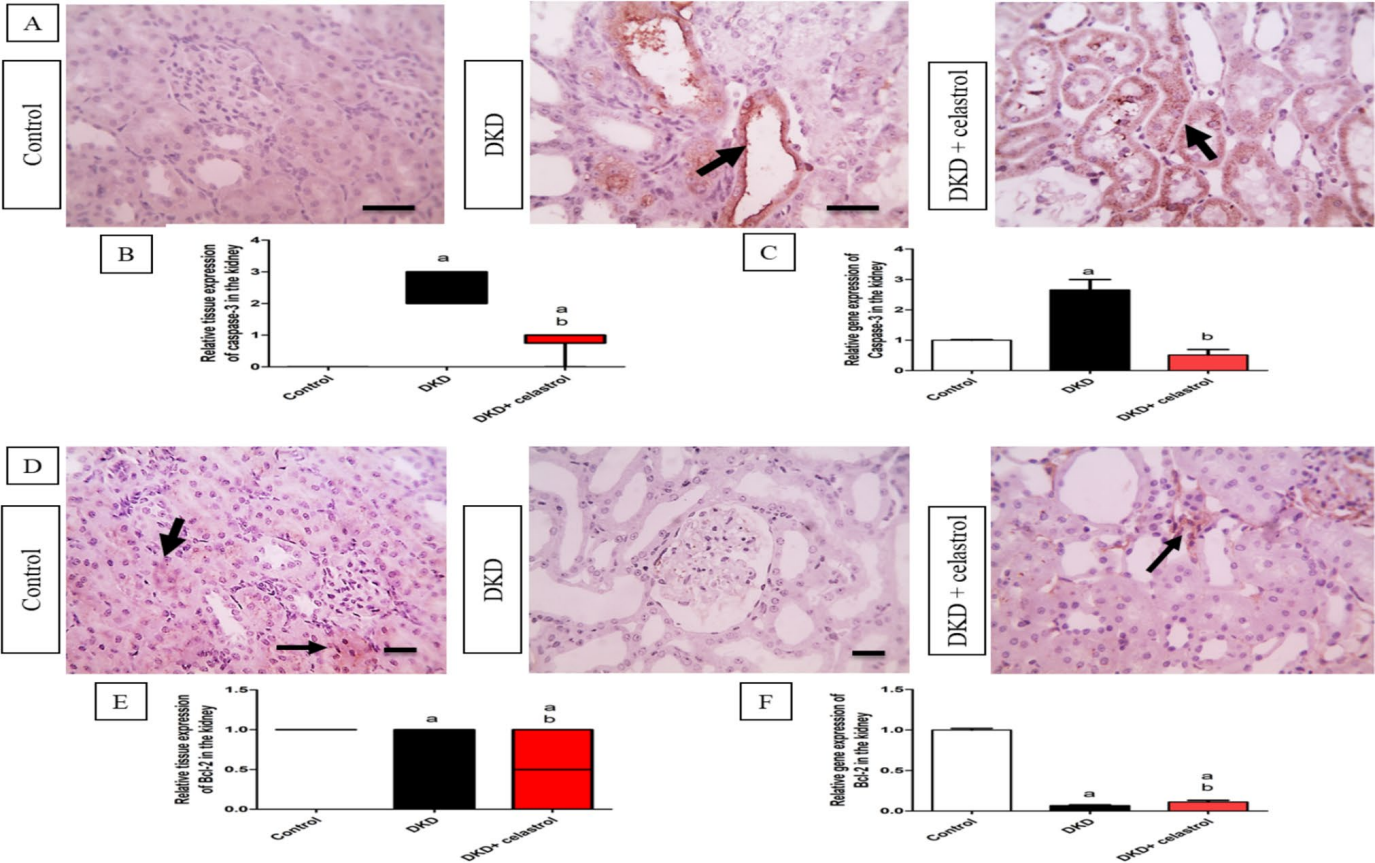
Effects of celastrol (1.5 mg/kg/day, orally) on gene and tissue expressions of caspase-3 and Bcl-2 in a rat model of diabetic kidney disease (DKD) induced by a single intraperitoneal injection of 55 mg/kg streptozotocin. (**A**) Microscopic pictures of immunostained renal sections against caspase-3 show no expression in the control group and a strong positive brown tubular reaction in the DKD group. However, sections from celastrol-treated rats show a moderate positive brown expression in some tubules. × : 400 bar 50. (**B**) Quantitative analysis of caspase-3 tissue expression. Data are expressed as the median of IHC score against caspase-3 analyzed by the Kruskal–Wallis test followed by Dunn’s test. (**C**) The relative gene expression of caspase-3 analyzed by real-time RT PCR. Data expressed as mean ± SE. a = significant at *P* < 0.05 versus control group, b = significant at *P* < 0.05 versus DKD group. (**D**) Microscopic pictures of immunostained renal sections against Bcl-2 show a diffuse mild positive brown tubular expression in the control group, a negative expression in the DKD group, and a mild brown tubular reaction in the celastrol-treated group. × : 400 bar 50. Solid arrows point to positively stained tubules. (**E**) Quantitative analysis of Bcl-2 relative tissue expression. Data are presented as the median of IHC score against Bcl-2 analyzed by the Kruskal–Wallis test followed by Dunn’s test. (**F**) The relative gene expression of caspase-3 analyzed by real-time RT PCR. Data expressed as mean ± SE. a = significant at *P* < 0.05 versus control group, b = significant at *P* < 0.05 versus DKD group

In contrast to the control group, the DKD group demonstrated a 2.7-fold increase in the expression of the proapoptotic gene (caspase-3) in the kidneys, and a 0.93-fold reduction in the antiapoptotic gene (Bcl-2) expression inside the kidneys (Fig. 5C and F). When contrasted with the control group, these outcomes are comparable to the immunohistochemistry findings for the expression of Bcl-2 and caspase-3. The proapoptotic caspase-3 gene was significantly downregulated by about 0.8-fold in the kidneys of the celastrol-treated rats, while the antiapoptotic Bcl-2 gene was upregulated by approximately 0.64 fold (Fig. 5C and F). The immunohistochemical study validated these findings, as rats treated with celastrol exhibited reduced caspase tissue expression compared to the DKD group. In contrast, the administration of celastrol resulted in a significant increase in the tissue expression of the antiapoptotic gene Bcl-2, as compared to the DKD group (Fig. 5A, B, D, and E, respectively).

### Celastrol effect on renal expression of autophagic markers

**Fig. 6:**
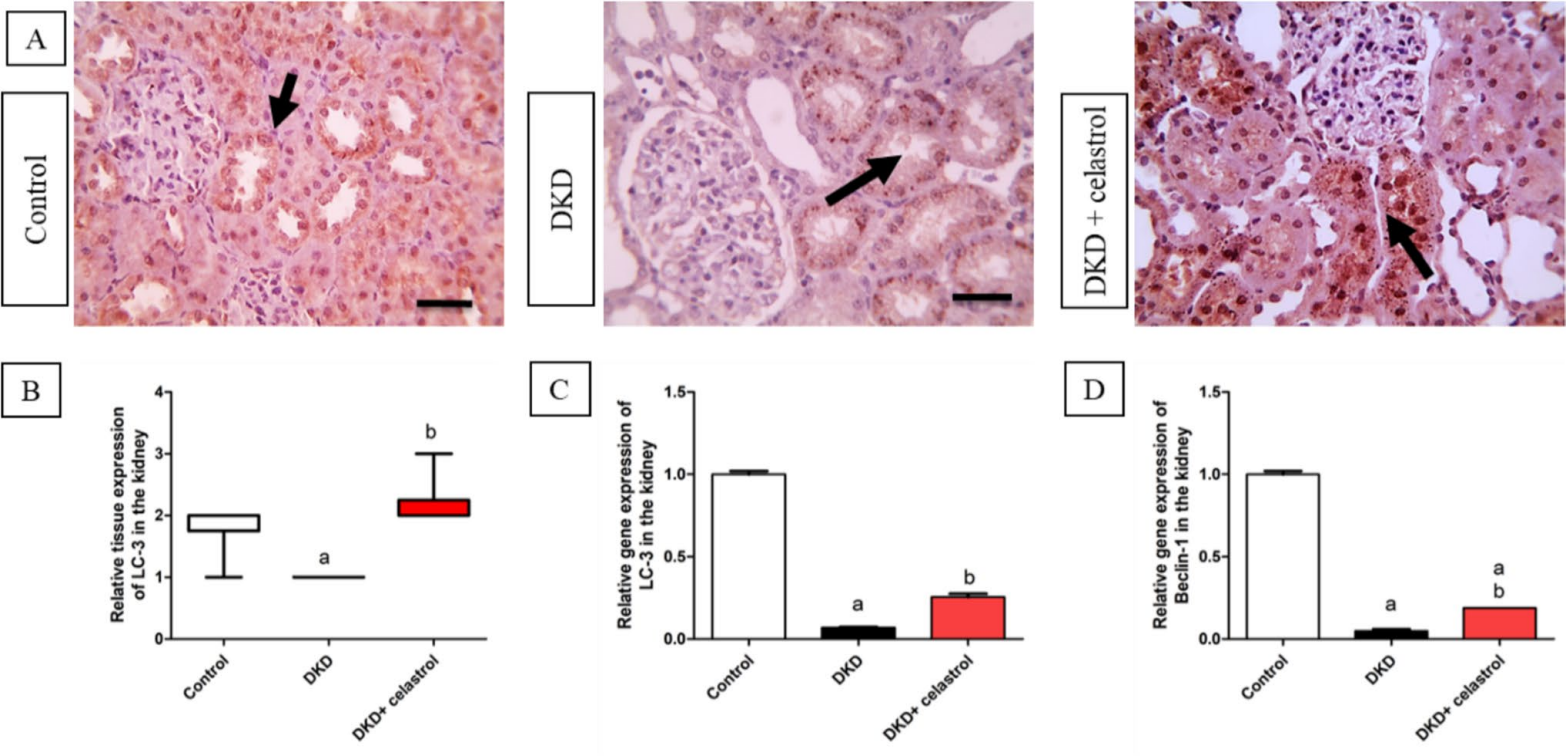
Effects of celastrol (1.5 mg/kg/day, orally) on relative tissue expression of LC-3 (**A**, **B**), relative gene expression of LC-3 (**C**), and Beclin-1 (**D**) in a rat model of diabetic kidney disease (DKD) induced by a single intraperitoneal injection of 55 mg/kg streptozotocin. Data are presented as Mean ± SEM (**C**, **D**). Number of animals in each group = 8. (**A**) Microscopic pictures of immunostained renal sections against LC-3 show a strong positive brown expression in almost all tubules in the control group. In contrast, the DKD group shows a weak positive brown expression in some tubules. However, sections from the celastrol-treated group show a moderate positive brown tubular reaction. × : 400 bar 50. Black arrows point to positively stained tubules. (**B**) Quantitative analysis of LC-3 relative tissue expression. Data are presented as the median of IHC score against LC-3 analyzed by the Kruskal–Wallis test followed by Dunn’s test. a = significant at *P* < 0.05 versus control group, b = significant at *P* < 0.05 versus the DKD group

In comparison to the control group, the DKD group’s renal gene expression of LC-3 and Beclin-1 was significantly lower, with around 0.93 and 0.95 folds, respectively (Kebede et al. 2021). On the other hand, Benclin-1 and LC-3 gene expression in the kidneys increased significantly as a consequence of using celastrol, with a fold change of about 2.6 and 2.7, respectively, compared to the DKD group (Fig. 6C and D). The previous results were confirmed by immunological investigation of LC-3 tissue expression. In comparison to the control group, this research showed a substantial decrease in LC-3 tissue growth in the DKD group. Rats given celastrol, on the other hand, showed substantially more LC-3 tissue expression than the DKD group, as seen in Fig. 6A and B.

### Celastrol impact on renal gene expression of inflammation and fibrosis markers

**Fig. 7:**
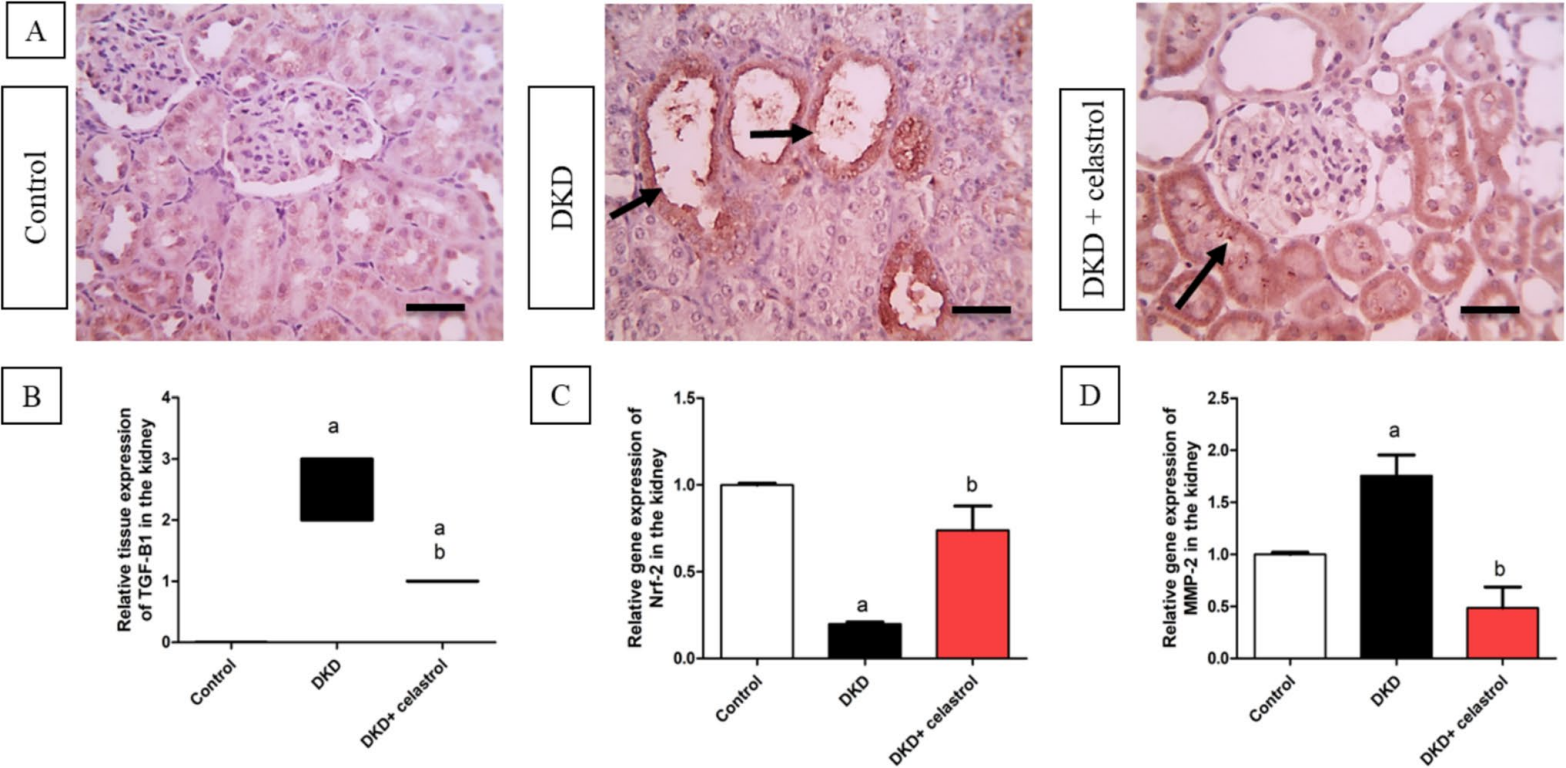
Effects of celastrol (1.5 mg/kg/day, orally) on relative tissue expression of transforming growth factor beta-1 (TGF-β1) (**A**, **B**), relative gene expressions of nuclear factor erythroid 2-related factor-2 (Nrf-2) (**C**), and matrix metalloproteinase-2 (MMP-2) (**D**) in a rat model of diabetic kidney disease (DKD) induced by a single intraperitoneal injection of 55 mg/kg streptozotocin. Data are presented as Mean ± SEM (**C**, **D**). Number of animals in each group = 8. (**A**) Microscopic pictures of immunostained renal sections against TGF-β1 show no expression in the control group, a strong positive brown tubular reaction in the DKD group, and mild positive brown tubular reaction in a few tubules of the celastrol-treated group. Black arrows point to positively stained tubules. High magnification × : 400 bar 50. (**B**) Quantitative analysis of TGF-β1 expression. Data are expressed as the median of IHC score against TGF-β1 analyzed by the Kruskal–Wallis test followed by Dunn’s test. a = significant at *P* < 0.05 versus control group. b = significant at *P* < 0.05 versus DKD group

DKD group exhibited substantially decreased Nrf-2 relative renal gene expression by approximately 0.8 folds compared to controls. The kidneys’ Nrf-2 gene expression was significantly upregulated after receiving celastrol, about 2.7 times higher than the DKD group (Fig. 7C). MMP-2 renal relative gene expression, on the other hand, was significantly increased in the DKD group—roughly 0.75 times higher than in the control group. When compared to the DKD group, the injection of celastrol caused the kidneys’ MMP-2 gene expression to decrease by about 0.72-fold, as shown in Fig. 7D. This result is consistent with the results of the immunohistochemical study, which revealed that the DKD group had a considerably greater tissue expression level of the fibrotic marker TGF-β1 than the control group did. In contrast, treatment with celastrol caused a considerably declined TGF-β1 tissue expression than the DKD group (Fig. 7A and B).

## Discussion

DKD is﻿ a common consequence of DM that often leads to ESRD and increased mortality rates (Kebede et al. 2021). Although there are treatment medications that may postpone the start of DKD, the use of herbal medicine to stop the onset of these problems has attracted a lot of attention (Mestry et al. 2017). Celastrol, a traditional Chinese herb derived from *Tripterygium wilfordii*, is an antifibrotic, anti-inflammatory, antioxidant, and antiapoptotic agent (Liu et al. 2021). Numerous studies have revealed that celastrol can provide protection against ischemia-induced injuries as well as renal injuries in db/db mice (Chu et al. 2014; Kim et al. 2013). Therefore, we sought to evaluate the potential protective effect of celastrol on kidney damage and explore the possible molecular pathways involved in an in vivo model of DKD in rats, with a specific emphasis on miRNA-192-5P and miRNA-21-5P.

After administering a single intraperitoneal dosage of STZ (55 mg/kg) to induce diabetes (Kumar et al. 2013), the DKD group demonstrated persistent hyperglycemia associated with significant elevation in creatinine, urea, 24-h urinary total protein, and microalbuminuria, along with decreased creatinine clearance. These outcomes are in line with the histological analysis, which showed disorganized architecture with obvious abnormalities, including interstitial edema, intervening fibrosis, and amplification of Bowman’s gap.

Our results showed that celastrol protects against DKD progression by improving glycemic control, anti-inflammatory effects, and antioxidant effects. These findings align with a prior study, which revealed that celastrol attenuated DKD progression in a rat model of type 2 diabetes (Nie et al. 2020). Nevertheless, the celastrol effect on miRNA-21-5P and miRNA-192-5P expression, as well as its correlation with autophagy, apoptotic, proinflammatory, and fibrotic pathways, has not yet been studied in a murine model of DKD.

In this study, celastrol significantly reduced HbA1c and blood glucose levels, aligning with other studies (Kim et al. 2013; Zhou et al. 2023). Renal function tests such as serum creatinine, urea, 24-h urinary total protein, and microalbuminuria were significantly decreased in celastrol-treated rats. The use of celastrol also reduced the histological alterations. Therefore, this study demonstrated the potential of celastrol to prevent DKD progression.

Recen﻿t﻿ studies h﻿ave demonstrated the potential of celastrol in ameliorating insulin resistance, particularly in type 2 diabetes mellitus (Zhan et al. 2018). Celastrol regulates glucose metabolism through its antioxidant and anti-inflammatory mechanisms, while also restoring key insulin signaling pathways (Nie et al. 2020). Although much of the existing literature focuses on type 2 diabetes, our study shifts attention to a type 1 diabetes model, where celastrol has shown promising effects in reducing oxidative stress and inflammation—two primary contributors to insulin resistance (Kim et al. 2013). In addition, celastrol’s inhibition of TXNIP (thioredoxin-interacting protein) further underscores its potential in mitigating these processes. Previous research using the Intraperitoneal Glucose Tolerance Test (IPGTT) has confirmed that celastrol significantly improves insulin sensitivity, highlighting its promise in glycemic management (Venkatesha and Moudgil 2016; Zhou et al. 2023).

Hyperglycemia can induce oxidative stress through various mechanisms, like the generation of the glycation end product, as well as activating alcohol pathways (Yaribeygi et al. 2019). DKD has been associated with oxidative stress and excessive free radical production. Oxidative species have been identified as a major contributor to the development of DKD in experimental models of diabetes (Sagoo and Gnudi 2018; Zhang et al. 2018). Therefore, the administration of antioxidants has been shown to alleviate the harmful effects of DKD (Zhang et al. 2018).

The current results revealed that celastrol administration restored TAC and CAT serum levels near the normal value, along with a concurrent decrease in the lipid peroxidation marker (MDA), demonstrating celastrol’s antioxidant effect. Our findings concur with previous research demonstrating celastrol’s ability to ameliorate DKD through antioxidant effects (Nie et al. 2020; Divya et al. 2016).

These antioxidant effects may be mediated through the increased expression of Nrf-2 in the celastrol-treated group, which is known as one of the most defender mechanisms that counteract oxidative stress. Nrf-2 regulates phase II detoxification enzymes, intracellular antioxidants, and other proteins detoxifying vital organisms and neutralizing ROS to regulate cellular redox homeostasis and enhance the survival of cells (Tonelli et al. 2018).

Furthermore, micro, small, single-stranded, RNA molecules that are not coding with an average length of 22 sequences are referred to as miRNAs. They specifically control target gene transcription by affecting with the integrity of mRNA and preventing the translation of mRNA. They also control destination gene expression through inadequate conjugation to the target transcript’s 3′-UTR region (Bartel 2004).

Many studies showed that miRNA imbalance has a more significant role in DKD progression (Deshpande et al. 2018; Li et al. 2019). Recently, research has demonstrated that miRNA-21-5p is one of the most crucial microRNA molecules implicated in renal fibrosis and with elevated levels in kidney tissues (Tang et al. 2019; Zhang et al. 2009).

Additionally, miRNA-21-5p was proven to cause fibrosis and renal injury by interfering with the cell cycle and causing mesangial hypertrophy since miRNA-21-5p was reported to downregulate TGF-β1signalling pathways (Sankrityayan et al. 2019). Moreover, (Hu et al. 2016) illustrated that miRNA-21-5p inhibited the expression of metalloproteinase-3 (TIMP3) tissue inhibitor and enhanced the expression of MMP-2.

MMPs serve as vital biophysical moderators of glomerular extracellular matrix (ECM) breakdown since they are normally controlled by TGF-β1 (Pourheydar et al. 2021). The prognosis of DKD and kidney failure depends on MMP-2 (Dejonckheere et al. 2011). Increased MMP-2 activity is seen in the early stages of CKD. Fibrosis may occur due to alterations in the phenotypic of renal tubular epithelial cells, type IV collagen degradation in the renal basement membrane, excessive production of TGF-β1, and inflammation of the glomerular membrane (Cheng et al. 2017).

Our results showed that rats treated with celastrol had a reduced expression of miRNA21-5p in their kidney tissues, along with a reduction in the expression of MMP-2 in the kidneys. This work is the first known investigation into the impact of celastrol on the renal expression of miRNA-21-5p and MMP-2.

According to research (Kato et al. 2007), both the glomeruli of diabetic mice and mouse mesangial cells exposed to TGF-β had higher levels of miRNA-192-5p. This elevation of miRNA-192-5p contributed to an increase in collagen accumulation in mesangial cells. Additionally, it was shown that suppressing miR-192 reduced renal fibrosis and enhanced proteinuria in a mouse model of DKD.

miRNA-21-5p and miRNA-192-5p have been linked to fibrosis, inflammation, and apoptotic pathways that cause kidney damage in the setting of DKD, especially through processes involving podocytes. Previous studies have demonstrated the therapeutic potential of targeting miRNA-21-5p and miRNA-192-5p in DKD. For instance, silencing miRNA-21-5p in cultured podocytes has been shown to reduce fibrosis and inflammation, highlighting its role in the pathogenesis of DKD (Dhas et al. 2023). Similarly, miRNA-192-5p knockdown in podocytes has been associated with a reduction in renal fibrosis and improvement in proteinuria in diabetic models (Putta et al. 2012). These findings suggest that targeting these microRNAs could be a promising strategy for mitigating kidney damage, underscoring their relevance as therapeutic targets in DKD.

Our study showed that celastrol had renoprotective effects by inhibiting miRNA-21 and miRNA-192 and their downstream pathways. Celastrol-treated rats displayed a downregulation in the renal expression of miRNA-192. Moreover, immunohistochemistry examination revealed a downregulation in TGF-β1 renal tissue expression than in the DKD group, suggesting that celastrol may have a renoprotective effect by inhibiting miRNA-21 and miRNA-192 and their downstream targeting pathways.

Hyperglycemia may induce apoptosis of renal cells leading to glomerular injuries as well as keratinocyte depletion, which is linked to glomerular structural damage and proteinuria in DKD (Zhang et al. 2022). On the contrary, decreased autophagy has been linked with the pathogenesis of DKD, as downregulated autophagic activity was observed in the podocyte of diabetic kidney, impairing the glomerular filtration barrier (Erekat 2022).

Autophagy and apoptosis are both cellular degradation pathways crucial for tissue homeostasis (Su et al. 2013). A family of Bcl2-related proteins includes negative and positive apoptotic regulators. Bcl-2 is antiapoptotic, while BAX is proapoptotic (Habib 2013). Our study showed that treatment with celastrol significantly increased the renal gene and tissue expression of the antiapoptotic marker, Bcl-2, along with a decreased renal gene and tissue expression of the proapoptotic marker, caspase-3, compared with the DKD group.

In DKD, NOX4 levels are elevated, leading to an increase in reactive oxygen species (ROS) and, consequently, oxidative stress, which exacerbates cellular damage and fibrosis in renal tissues (Lee et al. 2020). Our study demonstrated that celastrol effectively reduced NOX4 expression, thereby lowering ROS levels and mitigating oxidative stress. This reduction in oxidative stress likely contributes to the observed renoprotective effects of celastrol, positioning it as a promising therapeutic candidate for conditions marked by excessive ROS production and NOX4 activity. The inclusion of 8-hydroxydeoxyguanosine (8-OhDG) and the RNA expression of p47 phagocyte oxidase (p47phox) and NOX2 in the glomeruli have been extensively studied in relation to oxidative stress and kidney disease. 8-OhDG, a well-known biomarker for oxidative DNA damage, has been shown to be associated with mortality in chronic kidney disease, independent of inflammation (Dai et al. 2019). Furthermore, the expression of p47phox and NOX2, key components of the NOX complex, has been implicated in the pathogenesis of DKD and other forms of kidney injury. These markers play an essential role in glomerular injury and oxidative stress, which are central to the progression of kidney damage (Wang et al. 2015).

The LC3 protein is crucial for autophagy because it plays a vital role in elongating, maturing, and fusing autophagosomes (Gonzalez et al. 2021). Beclin-1 has a significant impact on the mechanism of autophagy. This is a part of the complex known as phosphatidylinositol-3-kinase, which helps vesicles travel and triggers the autophagy process. Beclin-1 dysfunction has been linked to several conditions, including cancer, diabetes, and neurodegenerative illnesses (Naguib et al. 2021).

The use of flow cytometry in DKD has facilitated insight into cellular mechanisms, particularly in analyzing apoptosis and signaling pathways. For instance, study (Liu 2023) examined IL-33/ST2L signaling pathway in DKD used flow cytometry to assess apoptosis in glomerular endothelial cells, showing that in diabetes mice, IL-33 therapy decreased endoplasmic reticulum stress and cellular death.

According to the present study, as compared to the DKD group, celastrol treatment raised the renal gene expression of LC-3B and Beclin-1. This discovery was validated by the results of the immunohistochemistry, which revealed that rats treated with celastrol had much higher tissue expression of LC-3B than the DKD group, indicating the protective impact of celastrol on the kidneys. This discovery may be linked to the heightened activation of an autophagic pathway, accompanied by the suppression of the apoptotic pathway. The research findings showed that celastrol enhanced the renal gene expression of LC-3B in DKD (Nie et al. 2020).

Apoptosis is a crucial process in the progression of DKD and it is important to clarify which renal cell types are undergoing cell death. Previous studies have shown that podocyte apoptosis plays a significant role in DKD, as podocytes are highly susceptible to apoptosis under diabetic conditions. This contributes to glomerular damage and proteinuria, both hallmarks of DKD. Double staining techniques are essential to identify and localize apoptotic cells within the kidney to distinguish the specific cell types involved. Podocyte apoptosis is commonly observed in DKD, as podocyte cell death is a key factor in the development and progression of the disease (Mima et al. 2020).

## Conclusion

The current investigation highlights the renoprotective effects of celastrol in DKD. The findings demonstrate that celastrol effectively ameliorates kidney dysfunction through its potent antioxidant, anti-inflammatory, and antifibrotic actions. Specifically, celastrol’s ability to downregulate miRNA-21-5p and miRNA-192-5p expression contributes to the reduction of renal injury and fibrosis, underscoring the therapeutic potential of targeting these microRNAs in DKD.

Furthermore, celastrol induces autophagy, as evidenced by increased LC-3 and Beclin-1 expression, while also inhibiting apoptosis, as reflected by the modulation of Bcl-2 and caspase-3 expression. These mechanisms collectively enhance renal function and protect against the progression of DKD. Given the compelling evidence from this study, celastrol emerges as a promising natural therapeutic candidate for the management of DKD, offering a new avenue for the development of treatments that target molecular pathways involved in the disease. Future clinical studies are warranted to further explore its efficacy and applicability in human patients with DKD.

## Supplementary Information

Below is the link to the electronic supplementary material.

ESM 1 (PNG 241 KB)

High Resolution Image (TIF 322 KB)

## Data Availability

All source data for this work (or generated in this study) are available upon reasonable request.

## Abbreviations

Atg8: Autophagy-related gene 8
CAT: Catalase
CKD: Chronic kidney disease
DM: Diabetes mellitus
DMSO: Dimethyl sulfoxide
DKD: Diabetic kidney disease
ECM: Extracellular matrix
ESRD: End-stage renal disease
HbA1c: Glycated hemoglobin
HPRT-1: Hypoxanthine-guanine phosphoribosyltransferase 1
IP: Intraperitoneally
LC-3: Microtubule-associated protein 1A/1B-light chain 3
LNA: Locked nucleic acids
MDA: Malondialdehyde
miRNAs: MicroRNA
MMP-2: Matrix metalloproteinase-2
Nrf-2: Nuclear factor erythroid 2–related factor 2
NOX4: NADPH oxidase 4
PI3K: Phosphatidylinositol-3-kinase
RCN: Relative copy number
STZ: Streptozotocin
p47phox: P47 phagocyte oxidase
TAC: Total antioxidant capacity
TGF-β1: Transforming growth factor β1
TIMP3: Tissue inhibitor of matrix metalloproteinase 3
8-OhDG: 8-Hydroxydeoxyguanosine
