# Supplementary figures and images for: Novel therapeutic target for diabetic kidney disease through downregulation of miRNA-192-5p and miRNA-21-5p by celastrol: implication of autophagy, oxidative stress, and fibrosis
Source: Naunyn Schmiedebergs Arch Pharmacol. 2024 Dec 19;398(6):6915–28. doi: 10.1007/s00210-024-03669-5 (PMC12125129; doi:10.1007/s00210-024-03669-5)

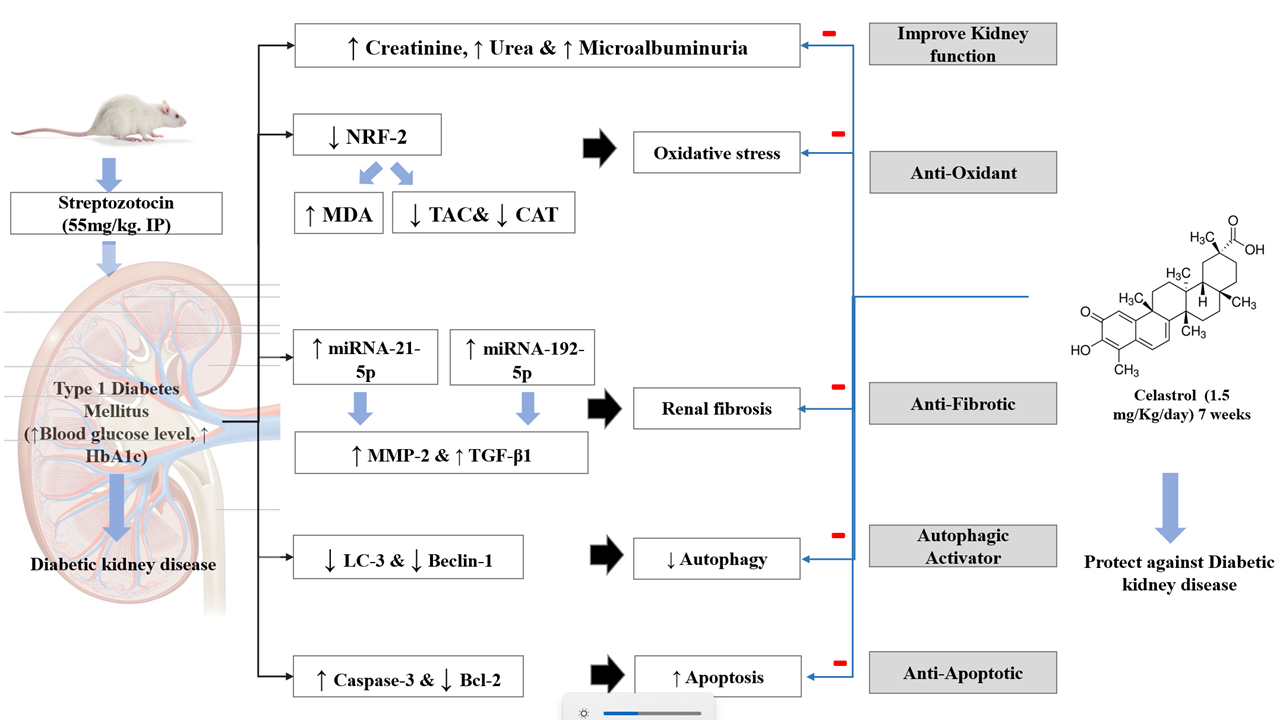

Supplement: Supplementary file 1 — (PNG 241 KB) [file 210_2024_3669_Fig8_ESM.png]

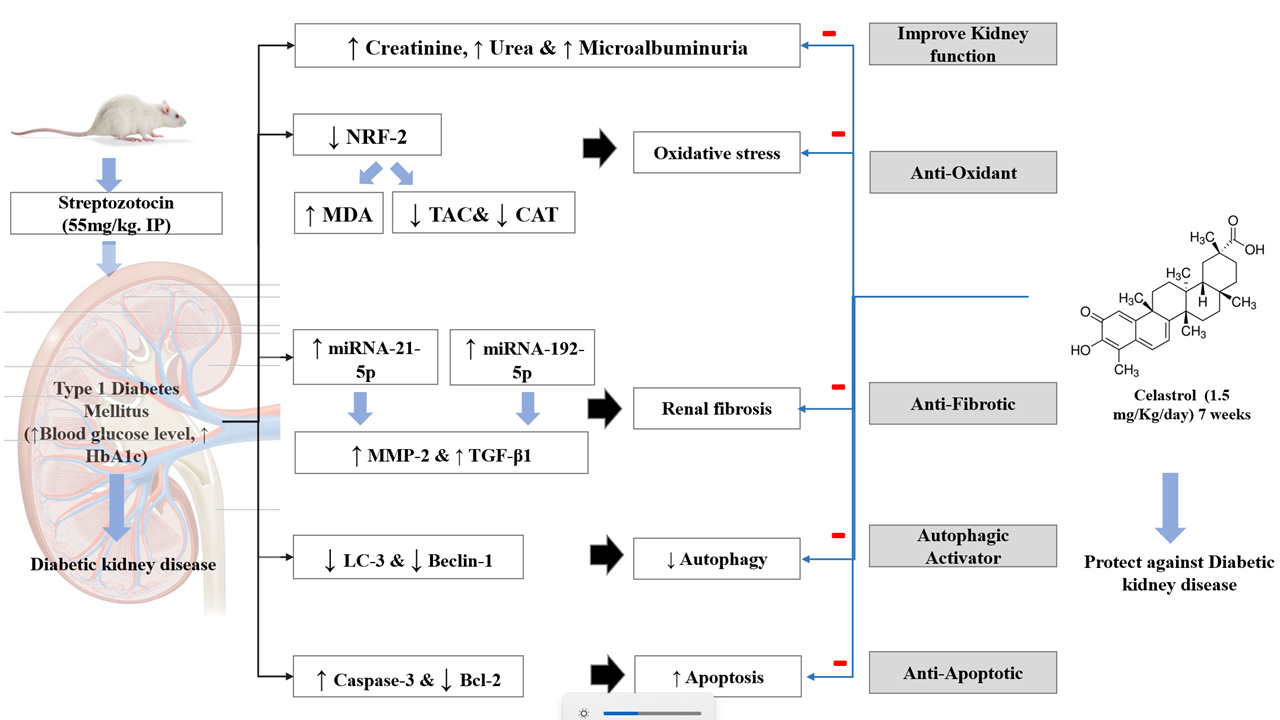

Supplement: Supplementary file 2 — High Resolution Image (TIF 322 KB) [file 210_2024_3669_MOESM1_ESM.tif]
